# A machine learning approach to identifying delirium from electronic health records

**DOI:** 10.1093/jamiaopen/ooac042

**Published:** 2022-05-24

**Authors:** Jae Hyun Kim, May Hua, Robert A Whittington, Junghwan Lee, Cong Liu, Casey N Ta, Edward R Marcantonio, Terry E Goldberg, Chunhua Weng

**Affiliations:** 1 Department of Biomedical Informatics, Columbia University, New York, New York, USA; 2 Department of Anesthesiology, Columbia University Medical Center, New York Presbyterian Hospital, New York, New York, USA; 3 Department of Epidemiology, Columbia University Mailman School of Public Health, New York, New York, USA; 4 Harvard Medical School, Boston, Massachusetts, USA; 5 Division of General Medicine, Department of Medicine, Beth Israel Deaconess Medical Center, Boston, Massachusetts, USA; 6 Division of Gerontology, Department of Medicine, Beth Israel Deaconess Medical Center, Boston, Massachusetts, USA; 7 Department of Psychiatry, Columbia University Irving Medical Center, New York, New York, USA

**Keywords:** delirium, Confusion Assessment Method for the Intensive Care Unit (CAM-ICU), electronic health records, logistic regression, machine learning model

## Abstract

The identification of delirium in electronic health records (EHRs) remains difficult due to inadequate assessment or under-documentation. The purpose of this research is to present a classification model that identifies delirium using retrospective EHR data. Delirium was confirmed with the Confusion Assessment Method for the Intensive Care Unit. Age, sex, Elixhauser comorbidity index, drug exposures, and diagnoses were used as features. The model was developed based on the Columbia University Irving Medical Center EHR data and further validated with the Medical Information Mart for Intensive Care III dataset. Seventy-six patients from Surgical/Cardiothoracic ICU were included in the model. The logistic regression model achieved the best performance in identifying delirium; mean AUC of 0.874 ± 0.033. The mean positive predictive value of the logistic regression model was 0.80. The model promises to identify delirium cases with EHR data, thereby enable a sustainable infrastructure to build a retrospective cohort of delirium.

## Lay Summary

Delirium is a commonly observed complication in hospitalized patients, especially with intensive care. While signs and symptoms of delirium could be observed and well managed during the hospital stay, less is known about the long-term complication of delirium after discharge. In order to monitor the long-term sequelae of delirium, the correct identification of delirium patients is crucial. Currently, the retrospective identification of delirium patients is limited due to the under-coding of delirium diagnosis in electronic health records. We proposed a simple machine-learning model to retrospectively identify patients who experienced delirium during their intensive care unit stay. The model could be used to identify missed delirium cases and the establishment of a delirium cohort for long-term monitoring and surveillance.

## INTRODUCTION

Delirium is a frequent complication among intensive care unit (ICU) patients, with its incidence ranging between 45% and 87% of all ICU patients.^1^^,^^2^ There are short-term and long-term impacts of delirium during an ICU stay on patients’ clinical outcomes. For instance, delirium is known to be associated with prolonged hospitalization, short- and long-term cognitive impairment, and increased healthcare costs.^3–5^ Delirium has also been associated with increased short- and long-term mortality.^6–8^ Nevertheless, according to ICU delirium practice guidelines, there still exists a significant research gap regarding the long-term outcomes of delirium.^3^ The establishment of retrospective delirium cohorts would be useful for long-term surveillance. However, the undercoding of delirium diagnoses and the burden of delirium screening in clinical practice inhibit the identification of delirium in the electronic health records (EHRs) and the establishment of retrospective cohorts.^9^^,^^10^ A number of delirium prediction models have been developed.^11^^,^^12^ Some developed multivariable models using 4–9 preoperative variables^13–15^ and other recent models used machine learning or deep learning algorithms including neural net.^16^^,^^17^ However, as stated, all of them used preoperative (or before admission) characteristics since the goal of these models was to predict ahead of time patients who may develop delirium after certain interventions such as hip surgery, in-patient admission, or ICU stay. Therefore, new diagnoses or drug prescriptions during hospitalization periods were not included in the clinical prediction model.

In contrast, the focus of this research was to retrospectively identify ICU patients who experienced delirium during hospitalization using a classification model. Considering that the occurrence of delirium would elicit a change in treatment pattern during hospitalization, the inclusion of variables recorded during hospitalization in the model could potentially increase the accuracy of the classification model. The study population included only patients who had been evaluated for delirium in the ICU using the standard Confusion Assessment Method for the Intensive Care Unit (CAM-ICU).

## METHODS

### Data

This study was approved by Columbia University Irving Medical Center (CUIMC) institutional review board and informed consent was waived. The dataset for model development included patients from either the Surgical or Cardiothoracic ICU with at least one CAM-ICU evaluation result during their ICU stay at New York Presbyterian Hospital (NYP)/CUIMC from January 30, 2018 to February 20, 2018. The CAM-ICU data were obtained as a part of a quality improvement project that aimed to improve recognition of delirium in the 2 ICUs. Raters received training (in the form of videos and a written manual) and performed CAM-ICU assessments on a convenience sample of patients. Inter-rater reliability was assessed using Gwet’s kappa in a sample of 15 patients and found to be high (0.9295, 95% confidence interval, 0.7689–1.000).^18^ If the patient was ever positive from at least one of the CAM-ICU evaluations, that patient was counted as having postoperative delirium.

The Medical Information Mart for Intensive Care III (MIMIC-III) dataset was used as an independent dataset for the validation of the model design.^19^ Patients with neonatal intensive care unit care history were removed from the full dataset. For a given patient, the first ICU visit was included in the validation in case a patient had multiple visit records. The history of delirium was identified from the CHARTEVENTS table of the MIMIC-III dataset. As in the CUIMC dataset, the patient was labeled as positive for delirium if that patient was ever positive from at least one of the CAM-ICU evaluations during their ICU stay.

### Model implementation

Following clinician guidance, we included the following features for model development: patients' age at the time of admission, sex, Elixhauser comorbidity index, diagnoses (eg, cirrhosis of liver, cerebral edema, etc), and drug prescription records. EHR data for these features were extracted from the Observational Medical Outcomes Partnership (OMOP) Common Data Model (CDM) version 5.3 formatted clinical data warehouse of NYP/CUIMC.^20^ A set of drug exposures of interest were preselected by the clinician. Selected drug exposures were grouped into drug classes including antibiotics, neuromuscular blocking agents, sedative analgesics, and vasoactive agents. The diagnosis and drug concepts that were extracted from the structured data are listed in Supplementary Table S1. In the CUIMC data, the Elixhauser comorbidity index was calculated with the records of diagnoses from 6 months prior to the admission to the date of admission. Age and Elixhauser comorbidity index were normalized to range from 0 to 1. Drug exposures and diagnoses were one-hot encoded (ie, 1 denotes the presence of a drug exposure or diagnosis at any time during the patient’s hospitalization, while 0 denotes no drug exposure or diagnosis) and represented as a vector.

We used 2 simple machine learning methods: logistic regression and multi-layer perceptron (MLP). The performance of each model was evaluated with 5-fold cross-validation. In each fold, the test set area under the receiver operating characteristic curve (AUC) was calculated, and the mean ± standard error of AUCs was presented. The validation set was not used in the model development. The learning rate (0.001 or 0.0001) and the number of epochs (20 or 30) were chosen using grid search based on the training loss. The number of epochs was set to 20. The learning rate was set to 0.001 for LR models and 0.0001 for MLP models. MLP models have a single hidden layer with 128 hidden units. The Adam optimizer was used in all models.^21^

Due to the limited sample size in our in-house dataset, we used calibrated approach as described in the research by Barak-Corren et al.^22^ The selection of the modeling algorithm and their features were done with CUIMC dataset. After choosing the machine learning model and features, the model was individually trained with the MIMIC-III data which resulted in the calculation of the new coefficients.

### Software

Python version 3.6.9 and Tensorflow version 2.3.1 were used.^23^ The code is available in the following GitHub repository: https://github.com/WengLab-InformaticsResearch/delirium. The R package comorbidity was used to calculate the Elixhauser comorbidity index.^24^

## RESULTS

We identified 76 patients who were admitted to NYP/CUIMC ICU and had evaluation results for the CAM-ICU for delirium from January 30, 2018 to February 20, 2018. Table 1 shows the characteristics of patients according to the delirium status. In the CUIMC dataset, their mean age was 63.4 ± 15.1 years and 59.2% (*n* = 45) of patients were male. The mean Elixhauser comorbidity index was 4.1 ± 3.2. The mean hospitalization duration was 28 ± 36 days. In total, 63.2% (*n* = 48) of patients were White. The CAM-ICU evaluation was conducted 123 times in 76 patients, with 1.6 times per patient on average. Among 76 patients, 17 patients (22.4%) had delirium in at least one of the CAM-ICU evaluations. Six out of 17 CAM-ICU positive patients and 10 out of 59 CAM-ICU negative patients had ICD-10-CM diagnoses of delirium, respectively.

**Table 1.** Characteristics of patients according to the delirium evaluation results

Variable	CUIMC dataset			MIMIC-III dataset			*P* value^a^
	Delirium positive	Delirium negative	Total	Delirium positive	Delirium negative	Total	
	(*n* = 17)	(*n* = 59)	(*n* = 76)	(*n* = 857)	(*n* = 2746)	(*n* = 3603)	
Age, mean (SD)^b^	68.6 (12.3)	61.9 (15.6)	63.4 (15.1)	65.8 (17.3)	63.6 (16.9)	64.1 (17.0)	.722
Male, *n* (%)	9 (52.9)	36 (61.0)	45 (59.2)	471 (55.0)	1516 (55.2)	1987 (55.1)	.556
Elixhauser comorbidity index, mean (SD)	5.2 (3.0)	3.8 (3.2)	4.1 (3.2)	4.8 (2.3)	3.8 (2.1)	4.0 (2.2)	.698
Hospitalization duration (days), mean (SD)	52.1 (49.9)	21.3 (28.4)	28.1 (36.4)	13.9 (11.8)	7.7 (7.7)	9.1 (9.3)	<.001
Race, *n* (%)							.773
White	10 (58.8)	38 (64.4)	48 (63.2)	538 (62.8)	1872 (68.2)	2410 (66.9)	
Black/African American	2 (11.8)	5 (8.5)	7 (9.2)	61 (7.1)	217 (7.9)	278 (7.7)	
Other/Unknown	5 (29.4)	16 (27.1)	21 (27.6)	258 (30.1)	657 (23.9)	915 (25.4)	
Delirium diagnosis code, *n* (%)^c^	6 (35.2)	10 (16.9)	16 (21.1)	152 (17.7)	104 (3.8)	256 (7.1)	<.001

CUIMC: Columbia University Irving Medical Center; MIMIC-III: Medical Information Mart for Intensive Care III; SD: standard deviation.

a *P* values for comparison between the CUIMC dataset and MIMIC-III dataset.

b Age over 89 was capped to 89 in the MIMIC-III dataset.

c ICD-10-CM F05 and R41.0 were used in the CUIMC dataset and ICD-9-CM codes 293.0, 293.1, 290.11, 290.3, 290.41, 291.0, 292.81, and 780.97 were used in the MIMIC-III dataset for delirium diagnosis code.

In the MIMIC-III dataset, 857 patients were positive for delirium among 3603 patients. The mean age was 64.1 ± 17.0 and 55.1% (*n* = 1987) of patients were male in the total cohort. The mean Elixhauser comorbidity index was 4.0 ± 2.2. The mean hospitalization duration was 9.1 ± 9.3 days. The most common race group was White (66.9%, *n* = 2410). The ICD-9-CM delirium diagnosis code was recorded in 7.1% of patients (*n* = 256). The CAM-ICU positive patients had more delirium diagnosis codes than the CAM-ICU negative patients: 17.7% (*n* = 152) in CAM-ICU positive versus 3.8% (*n* = 104) in CAM-ICU negative.

**Figure 1.**
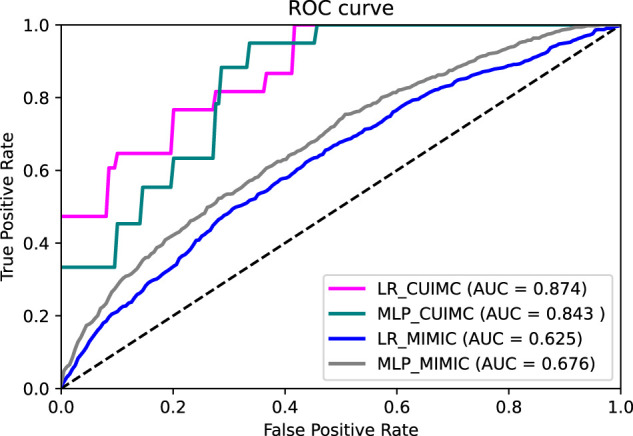
ROC curve of all models. ROC: receiver operating curve; CUIMC: Columbia University Irving Medical Center; MIMIC: Medical Information Mart for Intensive Care; LR: logistic regression; MLP: multi-layer perceptron.

**Figure 2.**
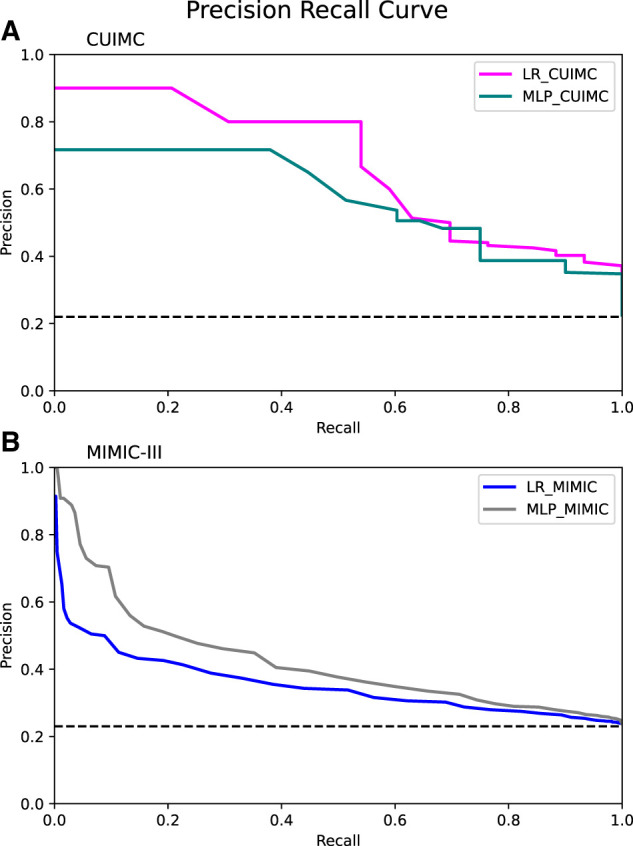
Precision–recall curve of the LR and MLP model in the CUIMC (A) and MIMIC-III datasets (B). CUIMC: Columbia University Irving Medical Center; MIMIC: Medical Information Mart for Intensive Care; LR: logistic regression; MLP: multi-layer perceptron. Dash lines show the proportion of positive cases among evaluated sample (0.22 and 0.23 for CUIMC and MIMIC-III dataset, respectively). All curves were above the baseline.

Figure 1 shows the receiver operating characteristic curve (ROC) of the LR and MLP model in the CUIMC and MIMIC-III datasets. The LR model showed the highest mean AUC (0.874 ± 0.033), followed by the MLP model (mean AUC 0.843 ± 0.053) with the CUIMC dataset. When the threshold of the LR model was 0.75, the mean positive predictive value (PPV), negative predictive value, sensitivity, and specificity were 0.80, 0.86, 0.54, and 0.95, respectively. When the models were applied to the MIMIC-III dataset, the AUC was 0.625 ± 0.017 for the LR model and 0.676 ± 0.017 for the MLP model. Among the evaluated features, the use of neuromuscular blocking agents such as rocuronium and cisatracurium during hospitalization are associated with a higher likelihood of being CAM-ICU positive the most. Following the use of neuromuscular blocking agents, the diagnosis of acute hypoxemic respiratory failure and hydronephrosis were considered as important features for classification. Figure 2 shows the precision–recall curve of all evaluated models. The precision–recall curves of the LR and MLP models were well above the baseline of 0.22 (proportion of positive cases among evaluated samples in the CUIMC dataset) in Figure 2A. In Figure 2B, the precision–recall curves of both models were above the baseline of 0.23.

## DISCUSSION

We applied the logistic regression and MLP model to classify ICU patients into delirium cases or controls using retrospective EHR data. We expect our models to have potential in the identification of patients with delirium in ICU while minimizing the human effort to manually review the accumulated patients’ records. Such a model would be useful for patient follow-up for better determining the long-term medical sequelae of ICU patients with a history of delirium.

Our classification model would be useful in the identification of missed patients with delirium and thereby augments the clinical diagnosis of delirium in combination with patient screening at bedside, albeit retrospectively. Our goal for developing this model was to help recruit patients who previously experienced delirium into a study to assess the long-term outcomes of the condition. In accordance with these goals, the classification model was tuned to achieve high PPV and specificity to increase the likelihood that candidates selected by the model truly had delirium. Patients with delirium could be underdocumented due to multiple reasons. First, factors including misinterpretation of patients' status, documentation errors, and education gap may lead to inappropriate documentation of CAM-ICU evaluation results.^25^^,^^26^ Second, in clinical practice, not all patients in the ICU receive the CAM-ICU evaluation for delirium, despite quality improvement efforts.^27^ Third, according to the study by Chanques et al, CAM-ICU had a sensitivity of 83%, and among the 108 CAM-ICU ratings, 7 cases were false negatives.^28^ For these reasons, a subset of delirium patients remains unevaluated, undocumented, or otherwise unidentified and hence lose a chance at being followed for the occurrence of complications of the delirium. Moreover, the evaluation of delirium was even more difficult in the recent COVID-19 pandemic.^29^ The COVID-19 survivors who had ICU care may possess high-risk for long-term cognitive sequelae, with a recent multi-center study finding a delirium prevalence of 54.9%.^30^

Among the features evaluated in the LR model, the use of neuromuscular blocking agents had the highest weights followed by the diagnosis of acute hypoxemic respiratory failure. Neuromuscular blocking agents such as cisatracurium or rocuronium are approved for endotracheal intubation, surgery, or mechanical ventilation. Patients with respiratory failure are likely to require ventilation support in the ICU. Although our model did not directly include the procedural codes, it is possible that these features were closely related to ventilation in the ICU. Mechanical ventilation was commonly included as a predictor in other delirium prediction models as well.^12^^,^^31^

This study has limitations. First, our model only included a small number of patients (*n* = 76) during the initial model development phase when compared to previous studies that developed machine learning algorithms to classify delirium. The patient data from the CUIMC used in this study were collected as a part of a quality improvement project in the ICU. The small amount of data can also restrict developing or applying complex machine learning models that have a lot of parameters to train. Therefore, the model was initially developed with the CUIMC data and the modeling algorithm and features were selected. Coefficients of the model were calculated with the MIMIC-III data while using the same model structure. With this approach we tried to test the generalizability of the model design at least. Second, the model was based on the Surgical or Cardiothoracic ICU data. The prediction of the model might not generalize into the patients in other ICU settings. Third, the portability of this model to other EHR data in other institutions should be further tested.

## CONCLUSION

We present a classification model that identifies patients with a delirium episode during their ICU stay using retrospective data. The classification model showed high accuracy with a mean AUC of 0.87. The model could be used in the retrospective identification of undiagnosed delirium cases and the establishment of a delirium cohort for long-term evaluation and surveillance.

## FUNDING

This study was sponsored by National Library of Medicine grant 5R01LM009886-11 and National Center for Advancing Clinical and Translational Science grants UL1TR001873 and 1OT2TR003434-01. The funders had no role in the design of this study and will not have any role during its execution, analyses, interpretation of the data, or decision to submit results.

## AUTHOR CONTRIBUTIONS

JHK wrote the manuscript; JHK, MH, TEG, RAW, and CW designed the research; MH provided the CAM-ICU evaluation results; MH selected the notes in the electronic health records for parsing; JL prepared the machine learning codes; JHK, JL, CL, and CNT refined the machine learning algorithm; JHK analyzed the data; CL contributed to the natural language processing of notes in the electronic health records; JHK, MH, RAW, JL, CL, CNT, ERM, TEG, and CW edited and approved the manuscript.

## ETHICS APPROVAL

This study was approved by Columbia University Irving Medical Center (CUIMC) institutional review board. Informed consent was waived since this study performs only retrospective records review.

## SUPPLEMENTARY MATERIAL

Supplementary material is available at *JAMIA Open* online.

## Supplementary Material

ooac042_Supplementary_Data
